# The Eye, Mind, Energy and Matter

**Published:** 1906-03

**Authors:** 


### BOOKS AND AUTHORS.

**The Eye, Mind, Energy and Matter.** By **Chalmers Prentice,** M.D. Duodecimo, pp. 131. Chicago: Published by the Author. 1905.

When two reviewers are required to properly present an opinion of a book, it must be either extremely good or hopelessly bad in quality. There was no considerable difficulty in placing this one just where it belonged. The author is frank enough to say its first half is intended for the general reader, the latter half for professional men. This in a measure accounts for the composite complexion of the review. One need not be surprised if he sees things after reading the book. The author is a versatile sort of genius, being his own publisher and bearing the honors of his own copyright. He springs a new idea in up to date book-making. He begins the book with a motto and follows that with a fable of home manufacture. The first half of the book, for the general reader, is an egotistic mass of poorly written, and illogical material, which is entitled to be rated for literary "general debility." The range of style is simply marvelous, going from light-hearted and fancy-free frippery about "be-autiful birds," to a heavy, harrowing disquisition about mind and energy. There is one thing, however, which the atuhor can conscientiously be commended for, and that is his choice of himself as his own publisher. Otherwise we fear the book would never have been issued from the press, hence we would be deprived of the pleasure of possessing it. Usually when one publshes a book at his own expense it is imprinted as a "limited edition for private circulation." That's what should have been done in this instance

It may be remarked with considerable reason that here is a man with one idea, who has written a whole book about it, a considerable portion of which is devoted to a wordy condemnation of fads. Apparently the author would have us believe that every disease may be cured by depriving the sufferer of acute vision, by applying prisms in a most arbitrary manner with the base out, regardless of the character of any muscular error that may exist or of whether or not such error does exist at all. If there be esophoria work the internal recti; while for hyperphoria the only treatment is to give the internal recti more exercise.

This condemnor of fads puts "fogging glasses" on his reason, mounts his little one-idea hobby and gallops serenely off to a ridiculous finish, when he states that the out-of-door treatment of pulmonary tuberculosis is entirely uninfluenced by climate; but that improvement under this plan is wholly due to the fact that the eyes are rested by gazing off into infinite distance. If this were true, none but highly myopic people could get any benefit from life in the Adirondacks or Canadian forests, where gazing at infinity is obviously impossible. We all know people who were not myopic, who did not wear "fogging glasses" but had tuberculosis, went to the Adirondacks where, in the thick forests, the longest distance they could see was perhaps a hundred feet, and came back cured. The best thing in the book is a remark about the word "idiosyncrasy." The author says "it means nothing and explains nothing." It would have been a good title for this book.
—N. W. W. L. B., Jr.

